# Dispensed drugs and multiple medications in the Swedish population: an individual-based register study

**DOI:** 10.1186/1472-6904-9-11

**Published:** 2009-05-27

**Authors:** Bo Hovstadius, Bengt Åstrand, Göran Petersson

**Affiliations:** 1 eHealth Institute, School of Pure and Applied Natural Sciences, University of Kalmar, SE-391 82 Kalmar, Sweden and Öhrlings PricewaterhouseCoopers, Box 179, SE-751 04 Uppsala, Sweden; 2 Apoteket AB and eHealth Institute, School of Pure and Applied Natural Sciences, University of Kalmar, SE-391 82 Kalmar, Sweden; 3 eHealth Institute, School of Human Sciences, University of Kalmar, SE-391 82 Kalmar, Sweden

## Abstract

**Background:**

Multiple medications is a well-known potential risk factor in terms of patient's health. The aim of the present study was to estimate the prevalence of dispensed drugs and multiple medications in an entire national population, by using individual based data on dispensed drugs.

**Methods:**

Analyses of all dispensed out-patient prescriptions in 2006 from the Swedish prescribed drug register. As a cut-off for multiple medications, we applied five or more different drugs dispensed (DP ≥ 5) at Swedish pharmacies for a single individual during a 3-month, a 6-month, and a 12-month study period. For comparison, results were also calculated with certain drug groups excluded.

**Results:**

6.2 million individuals received at least one dispensed drug (DP ≥ 1) during 12 months in 2006 corresponding to a prevalence of 67.4%; 75.6% for females and 59.3% for males. Individuals received on average 4.7 dispensed drugs per individual (median 3, Q1–Q3 2–6); females 5.0 (median 3, Q1–Q3 2–7), males 4.3 (median 3, Q1–Q3 1–6).

The prevalence of multiple medications (DP ≥ 5) was 24.4% for the entire population. The prevalence increased with age. For elderly 70–79, 80–89, and 90-years, the prevalence of DP ≥ 5 was 62.4, 75.1, and 77.7% in the respective age groups. 82.8% of all individuals with DP ≥ 1 and 64.9% of all individuals with DP ≥ 5 were < 70 years.

Multiple medications was more frequent for females (29.6%) than for males (19.2%). For individuals 10 to 39 years, DP ≥ 5 was twice as common among females compared to males. Sex hormones and modulators of the genital system excluded, reduced the relative risk (RR) for females *vs*. males for DP ≥ 5 from 1.5 to 1.4.

The prevalence of DP ≥ 1 increased from 45.1 to 56.2 and 67.4%, respectively, when the study period was 3, 6, and 12 respectively months and the corresponding prevalence of DP ≥ 5 was 11.3, 17.2, and 24.4% respectively.

**Conclusion:**

The prevalence of dispensed drugs and multiple medications were extensive in all age groups and were higher for females than for males. Multiple medications should be regarded as a risk in terms of potential drug-drug interactions and adverse drug reactions in all age groups.

## Background

The total sale of drugs has increased successively during the last decades [1,2]. The increase depends, among other things, on the introduction of new medications and on new medical recommendations to treat morbidity in higher ages. Moreover, drugs are also used to prevent health-related disorders among healthy individuals [3].

On a national level, the vast majority of all medications are dispensed drugs, but over-the-counter drugs (OTC), vitamins, herbal, alternative remedies, and in-hospital medications do all contribute to the total drug consumption.

The individuals' use of several different drugs, the so-called multiple medications, has also increased [4-6]. The use of many drugs may be rational for many individuals. However, multiple medications is also commonly associated with an irrational, excessive use of drugs, and are, concomitantly taken, a well-known risk factor in terms of patient's health [4,7,8]. Furthermore, the risk of drug-drug interactions and adverse drug reactions is expected to increase exponentially with the number of drugs [9].

Multiple medications may also result in an unnecessary health expenditure [8], directly due to redundant drug sales and indirectly due to the increased hospitalization caused by drug-related problems [10]. Drug-related problems are reported to cause between 10 and 20% of all emergency cases in hospitals and up to 20% of all admissions to hospitals of elderly patients [7,11].

Studies of multiple medications have mainly been based on small samples of elderly individuals admitted to hospitals or nursing homes [8]. A few studies have been based on population-based information [8,12,13], but some of these studies have also been limited to elderly individuals [14-17]. Therefore, it is uncertain to what extent multiple medications are relevant for other age groups too.

The establishing of the Swedish prescribed drug register in 2005 made it possible to use individual data to explore and analyse the utilisation of dispensed drugs and multiple medications for an entire national population. Furthermore, the register made it possible to compare different validated methods of estimating prevalence of multiple medications from large databases; 3-month prevalence [8], 6-month prevalence [18], and 12-month prevalence [19,20].

### Aim of the study

The aim of the present study was to estimate the prevalence of dispensed drugs and multiple medications in an entire national population, by using individual based data on dispensed drugs.

## Methods

To estimate the extent of multiple medications in a national population, we studied the individual based data of dispensed prescription drugs in the entire Swedish population between January 1, 2006 and December 31, 2006. The data was extracted from the Swedish prescribed drug register [13].

As a definition for the individual drug use, we applied one or more dispensed prescription drugs during a 12-month period (DP ≥ 1). The prevalence of drug use was consequently defined as the proportion of individuals who received one or more dispensed drugs during a 12-month period.

As a cut-off for multiple medications, we applied five or more dispensed prescription drugs during a 12-month period (DP ≥ 5). Since five or more drugs is one of the most commonly used definitions of polypharmacy and multiple medications [15,21], it might enable comparisons with other studies. Consequently, the prevalence of multiple medications was defined as the proportion of individuals who received five or more dispensed drugs during a 12-month period.

For comparison, we studied two shorter study periods; a 3-month period (1 Jan – 31 Mars 2006), and a 6-month period (1 Jan – 30 June 2006). Furthermore, we also calculated the prevalence when certain drug groups were excluded; Sex hormones, Antibacterials for systemic use, and Psycholeptics.

Another concept related to multiple medications is polypharmacy, which means the administration of many drugs at the same time or the administration of an excessive number of drugs [22]. The two terms are sometimes overlapping. The clinical implications of the two terms are that multiple medications, when measured as period prevalence, can include continuous use, when needed and short periods of drug use. Polypharmacy should only be related to concomitant drug use, but is also often measured as a period prevalence. Whether any number of dispensed drugs is relevant as a measure of polypharmacy in a clinical perspective is under debate [21,23]. In relation to polypharmacy, multiple medications (DP ≥ 5) is a period prevalence measurement, yielding a sum of the individual's dispensed drugs during a specific period without taking into account the concurrency or the appropriateness of the drug use.

The Swedish prescribed drug register is individual-based and contains data from dispensed out-patient prescriptions at all Swedish pharmacies from July 1, 2005, including multi-dose dispensed prescriptions and legal Internet sales. The registration is mandatory and the following data from the register were employed in our study: dispensed drug (substance), date of dispensing, age, gender, and a unique identifier (personal identification number) of the patient.

The prescribed drug register covers the entire Swedish population (patient identity data is missing for < 0.3% of all dispensed drugs) [13] and included in 2006 82% of all defined daily doses (DDD) distributed in Sweden. The register does not include data on OTC medications (13%), in-hospital medications (4%), and non-institutional care medications (1% of all DDD) [24]. The register is not complete for vaccines and for non-dose-dispensed drugs in nursing homes [13].

All data processing in our study was done anonymously without the personal identification number. Only gender and year of birth, originally embedded in the personal identification number, were recorded. The study population was stratified by gender and age (10-year classes). The results were compared to the number of individuals per gender and age group in the Swedish population.

Calculation of sums, frequencies and ratios were aggregated using Microsoft Excel (version 5.1.26).

The values applied were the number of individuals and the number of dispensed prescription drugs. The definition of drug was the chemical entity or substance comprising the fifth level in the Anatomical Therapeutic Chemical classification system (ATC). Epidemiological characteristics were defined and applied; prevalence – the proportion of individuals with five or more dispensed drugs in the Swedish population during 3-, 6-, and 12-month; relative risk, (RR) – the ratio between rates in two groups.

The proportion of total drug sales included in the register was assessed by comparing the data in the register with information on the total sales of medicines (prescriptions, OTC, and hospital sales) obtained from the National Corporation of Pharmacies (Apoteket AB).

The study was approved by the Regional Ethical Vetting Board in Linköping, Sweden.

## Results

### Prevalence of DP ≥ 1 and DP ≥ 5

Corresponding to 67.4% (6,146,679/9,113,257) of the entire population, 6.2 million individuals received at least one dispensed drug (DP ≥ 1) in 2006; 75.6% for females and 59.3% for males. A total of 2.2 million individuals, 24.4% of the entire population, received five or more different dispensed drugs (DP ≥ 5); 29.6% for females and 19.2% for males (Table 1).

**Table 1:** The prevalence of one or more (DP ≥ 1) and five or more (DP ≥ 5) dispensed drugs related to age and gender.

	DP ≥ 1			DP ≥ 5			RR for females	
			
	All	Females	Males	All	Females	Males	DP ≥ 1	DP ≥ 5
Age	n = 6,146,679	3,466,243	2,680,436	2,227,152	1,356,934	870,218		
0–9	57.3	55.8	58.8	6.6	5.7	7.3	0.9	0.8
10–19	49.0	58.4	40.1	6.2	8.3	4.1	1.5	2.0
20–29	58.4	76.4	41.2	9.9	15.2	5.0	1.8	3.1
30–39	62.5	75.3	50.3	13.9	19.7	8.4	1.5	2.3
40–49	64.3	73.3	55.6	18.4	23.8	13.2	1.3	1.8
50–59	75.8	82.5	69.3	30.2	36.3	24.3	1.2	1.5
60–69	80.1	83.6	76.5	42.3	46.8	37.7	1.1	1.2
70–79	90.8	92.3	89.0	62.4	65.5	58.7	1.0	1.1
80–89	94.5	95.1	93.5	75.1	77.3	71.6	1.0	1.1
90-	93.3	93.6	92.4	77.7	79.4	73.3	1.0	1.1

Total	67.4	75.6	59.3	24.4	29.6	19.2	1.3	1.5

The prevalence (%) of DP ≥ 1 and DP ≥ 5 related to age and gender and the relative risk (RR) for females vs. males with DP ≥ 1 and DP ≥ 5 in Sweden in 2006.

### Age and gender of individuals with DP ≥ 1 and DP > 5

**Figure 1:**
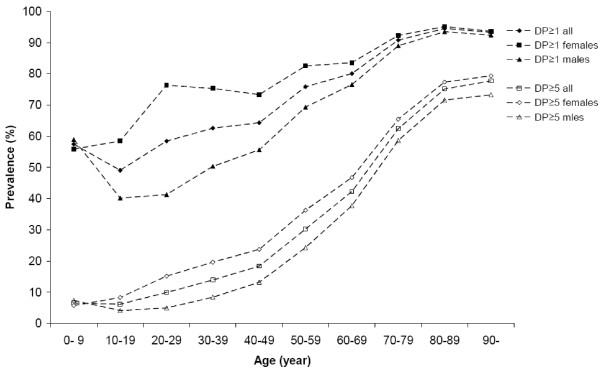
**The prevalence of one or more (DP ≥ 1) and five or more (DP ≥ 5) dispensed drugs**. The prevalence (%) of DP ≥ 1 and DP ≥ 5 related to gender and age groups in Sweden in 2006. Number of individuals with DP ≥ 1 = 6,146,679 (females = 3,466,243 and males = 2,680,436). Number of individuals with DP ≥ 5 = 2,227,152, (females = 1,356,934 and males = 870,218).

The prevalence of DP ≥ 1 was 57.3% in the age group 0–9 and 49.0% in the age group 10–19, subsequently increasing with age to 94.5% in the age group 80–89. DP ≥ 5 also increased with age, from 6.6% in the age group 0–9 to 77.7% in the age group 90 and above (Table 1) (Figure 1).

**Figure 2:**
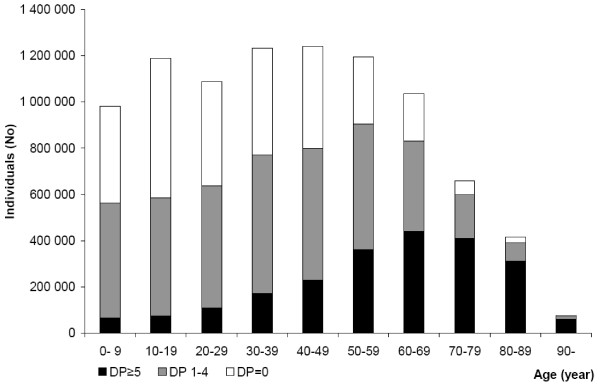
**The number of individuals with and without dispensed drugs related to age**. The total number of individuals related to age in Sweden 2006; no dispensed drugs (DP = 0), one to 4 dispensed drugs (DP1–4) and 5 or more dispensed drugs (DP ≥ 5). Number of individuals = 9,113,257.

82.8% (5,086,701/6,146,679) of all individuals, to whom at least one drug was dispensed, were less than 70 years of age. For all individuals, with five or more different drugs, 64.9% (1,446,062/2,227,152) were less than 70 years of age (Figure 2).

More females than males received at least one dispensed drug with an RR of 1.3 ((3,466,243/4,589,734)/(2,680,436/4,523,523)). More females than males also received DP ≥ 5 with an RR of 1.5 (Table 1).

The RR for females *vs*. males in terms of receiving five or more different dispensed drugs increased from 0.8, in the age group 0–9, to 3.1, in the age group 20–29, followed by the relative risk successively decreasing; from 2.3 in the age group 30–39 to 1.1 in the age groups 70 years and above (Table 1).

### The mean number of dispensed drugs specified according to gender and age

**Figure 3:**
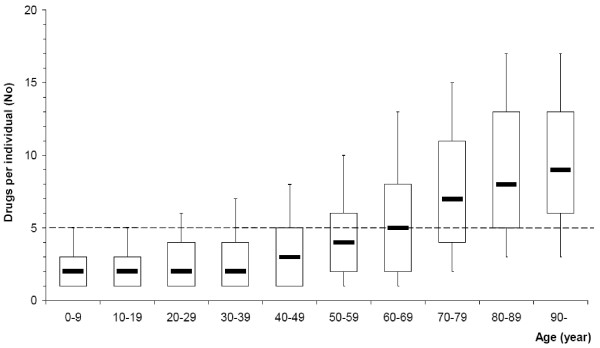
**The number of different dispensed drugs *per *individual related to age**. The number of different dispensed drugs *per *individual related to age in Sweden 2006 shown as box plots (median, Q1 and Q3 indicated). The 10th and 90th percentiles are shown at the end of the lines, (- - -) mark DP ≥ 5. Number of individuals = 6,146,679.

During the 12-month study period, the mean number of dispensed drugs for all individuals in Sweden receiving dispensed drugs was 4.7 (median 3, Q1–Q3 2–6) per individual; for females 5.0 (median 3, Q1–Q3 2–7), for males 4.3 (median 3, Q1–Q3 1–6) per individual. For elderly persons, 70 years and above, the mean number of dispensed drugs was 7.9 (median 7, Q1–Q3 4–11), 9.3 (median 8, Q1–Q3 5–13), and 9.7 (median 9, Q1–Q3 6–13) in the respective age groups; for children 0–9 the mean number of dispensed drugs was 2.4 (median 2, Q1–Q3 1–3) (Figure 3).

### The impact of the length of the study period on the prevalence of DP ≥ 1 and DP ≥ 5

**Figure 4:**
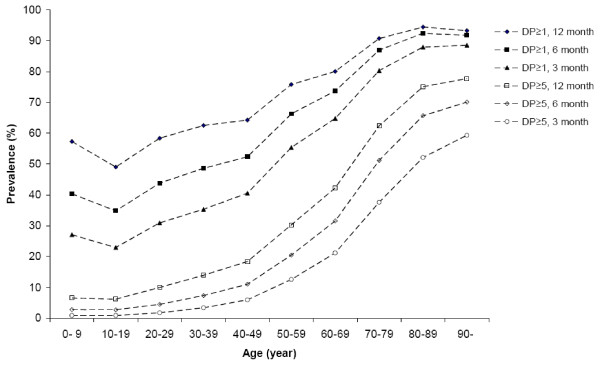
**The prevalence of one or more (DP ≥ 1) and five or more (DP ≥ 5) dispensed drugs in a 3-, 6- and 12- month study period**. The prevalence (%) of DP ≥ 1 and DP ≥ 5 in a 3-, 6- and 12- month study period in Sweden in 2006. Number of individuals with DP ≥ 1 in the 3-month period = 4,108,730, in the 6-month period = 5,117,817 and in the 12-month period = 6,146,679. Number of individuals with DP ≥ 5 in the 3-month period = 1,031,397, in the 6-month period = 1,569,180 and in the 12-month period = 2,227,152.

The prevalence of DP ≥ 1 and DP ≥ 5 increased with the length of the study period. With a study period of 3 and 6 months, the prevalence for DP ≥ 1 were 45.1 and 56.2%, respectively, and the prevalence of DP ≥ 5 were 11.3 and 17.2%, respectively (Table 2). The study period length had the greatest impact on the prevalence for the younger age groups but had a relative minor impact on the prevalence for the oldest age groups (Figure 4).

**Table 2:** The prevalence of five or more (DP ≥ 5) dispensed drugs with and without ATC G03^1^, J01^2 ^and N05^3^.

	All individuals DP ≥ 5				Females DP ≥ 5	
		
	All drugs	ATC G03 excluded	ATC J01 excluded	ATC N05 excluded	All drugs	ATC G03 excluded
Age	n = 2,227,152	2,131,961	1,997,440	2,111,966	1,356,934	1,262,497
0–9	6.6	6.6	3.6	6.5	5.7	5.7
10–19	6.2	5.2	4.3	5.9	8.3	6.4
20–29	9.9	8.3	7.5	9.1	15.2	11.8
30–39	13.9	12.5	11.2	12.8	19.7	16.7
40–49	18.4	17.4	15.9	16.8	23.8	21.8
50–59	30.2	28.9	27.4	28.3	36.3	33.5
60–69	42.3	41.1	39.4	40.4	46.8	44.4
70–79	62.4	61.6	60.0	60.2	65.5	64.1
80–89	75.1	74.7	73.2	72.7	77.3	76.7
90-	77.7	77.4	75.9	74.7	79.4	79.0

Total	24.4	23.4	21.9	23.2	29.6	27.5

The prevalence (%) of DP ≥ 5 for all individuals and for females, with and without ATC G03, J01, and N05 in Sweden in 2006.

^1 ^Sex hormones and modulators of the genital system

^2 ^Antibacterials for systemic use

^3 ^Psycholeptics

### The impact of Sex hormones, Antibacterials for systemic use and Psycholeptics on the prevalence of DP ≥ 5

When data on ATC G03 (Sex hormones and modulators of the genital system) were excluded, the prevalence of DP ≥ 5 for females was reduced from 29.6 to 27.5%. As a consequence, also the prevalence of DP ≥ 5 for all individuals was reduced, and the RR for DP ≥ 5 for females *vs*. males was reduced to 1.4 (Table 2).

When data on ATC J01 (Antibacterials for systemic use) were excluded, the prevalence of DP ≥ 5 for all individuals were reduced from 24.4 to 21.9%. In the age group 0–9, the exclusion of ATC J01 almost dimidiated the prevalence of DP ≥ 5, from 6.6 to 3.6%. In the age groups 70 and above, the exclusion of ATC J01 had a relatively minor effect on DP ≥ 5 (Table 2).

The exclusion of ATC N05 (Psycholeptics) had a marginal impact on the prevalence of DP ≥ 5 in all age groups except the age groups 20–29, 30–39, and 40–49 (Table 2).

## Discussion

We found that during one year, more then 2/3 of all individuals in a national population received at least one dispensed drug and that a considerable proportion, about 1/4, was exposed to multiple medications, a known potential risk factor for patients' health.

### Age and gender

Our findings reveal that multiple medications is not only a relevant issue regarding the elderly but also for other age groups. Thus, 2/3 of all individuals with multiple medications were younger than 70 years of age. Furthermore, there were more individuals with multiple medications in the age group 50–59 than in the age group 80–89, and almost four times as many in the age group 40–49 as in the age group 90 and above.

Our findings that the median number of dispensed drugs for all individuals, 70 years and above was 7, 8, and 9 in the respective 10-year age groups, are in line with previous reports [15,25].

Multiple medications, being more frequent for females than for males, may partly be explained by the use of sex hormones and modulators of the genital system (ATC G03) among fertile females [26]. When ATC G03 were excluded from our data, the prevalence of multiple medications declined in all age groups above 10 years, but the RR for females for DP ≥ 5 only decreased from 1.5 to 1.4. The increased RR among females *vs*. males for multiple medications is in line with previous studies [15,27] and may partly be explained by that females, of all ages, visit a doctor more often than males [28,29].

### The validity of dispensed drugs as an estimator of multiple medications

When dispensed drugs are used as an estimator of drug use and multiple medications, some conditions could cause both over- and underestimations. For a variety of reasons, a certain percentage of all drugs will never be used by patients, resulting in an overestimation of drug use when studying dispensed drugs [26]. On the reverse, the prevalence of drug use and multiple medications may be underestimated, as patients also use other medications than dispensed drugs. Additional sources, such as in-hospital medications, previously filled prescriptions (before the study period), OTC sales, herbal and alternative remedies, gifts and elicit Internet sales contribute to an underestimation of the total consumption of drugs. The absence of in-hospital medications in our data have different impact on different age groups, since the majority of the in-hospital medications is given to elderly individuals.

Among additional sources, OTC-drugs are of special interest, as previous studies have demonstrated a clear association between the use of prescription drugs and OTC drugs [10]. The vast majority of individuals over 65 use OTC drugs regularly [30] and different studies have shown that elderly people regularly use one OTC drug for every 2–3 prescribed drugs [30-32]. Applied on our data *e.g*., five dispensed drugs should correspond to a total use of seven drugs, OTC-drugs included.

Concomitantly taken multiple medications is a known risk factor for the patient's health. Many dispensed drugs are prescribed to be taken regularly. Some drugs, such as certain analgesics, are meant to be taken temporarily only when needed. Other drugs like antibiotics, are mostly intended to be taken periodically; a quarter of all individuals in Sweden in 2006 received one or more courses of treatment of ATC J01 (Antibacterials for systemic use). Periodically used drugs have different impact on the prevalence of multiple medications in different age groups. Antibacterials for systemic use had a huge impact on the prevalence of multiple medications in the age group 0–9, but only a minor effect on the prevalence in the age groups 70 and above. Antibacterials for systemic use were the most commonly prescribed drugs for individuals in the age group 0–9, but just one of many different used drugs for elderly with multiple medications.

Another source of underestimation of multiple medications in our data was generic duplication of dispensed drugs, recognised as a common problem in health care [4,7,33]. We did not evaluate the number of duplicates for each individual, but only calculated the number of dispensed drugs comprised of different substances. If the generic duplicate had been taken into account, it would have resulted in an even larger prevalence of multiple medications.

### Methods to estimate multiple medications on a national level

Data of drug utilisation and multiple medications may be available from the prescribers' medical records, pharmacy registers, or from the patient. The medical record may be preferable when to study the actual prescription orders, whereas data from pharmacies provides a better picture of what drug the patient actually received. Data collected from individuals may be closer to the true exposure of drugs, but are empirically associated with both intended and unintended memory failures.

In the present study, we choose a cumulative method [18] and counted all dispensed drugs, subsidized and non-subsidized, for all individuals in all ages during a 12-month period. Thereby, we compensated the monthly variation of dispensed drugs during a year, which in Sweden in 2006 varied by more than 20% between different months.

A 12-month study period also includes all females with prescribed sex hormones (ATC G03). Approximately three quarter of all females with ATC G03 receives these drugs, in contrast to other continually used drugs, for a 12-month period at one single pharmacy visit. A shorter study period, *e.g*. 3- or 6-month, will capture only a fraction of the number of females with ATC G03.

Our study shows that the length of a study period is essential for the estimation of the prevalence of the drug use and multiple medications, especially for the younger age groups. Compared to a study from Denmark based on a sample from a large regional database over dispensed drugs [8], we found a substantially higher prevalence of drug use and multiple medications in an entire national population. The difference between the results can partly be explained by differences in methods of estimation; in the Danish study were only subsidized prescription drugs and drugs with established DDD included. Other contributing explanations were that DDD per 1,000 inhabitants per day differs substantially between the countries [34], and also that there were 14 years between the two studies, 1994 and 2006. The continued increase in the use of drugs may therefore have influenced the estimates of the prevalence of DP ≥ 1 and DP ≥ 5. Compared to a study, based on interviews with a sample of elderly in Sweden [35], our observed prevalence for the age group was substantially higher. Possible explanations to the difference might be a minor difference in the definition of multiple drug use, and also sample and interview bias. The presented prevalences in three other studies from Sweden, based on individual register data of dispensed drugs [13-15], were close to our findings, with the reservation that the observed "polypharmacy" was not explicitly defined in one of the studies [14].

### Clinical relevance and implications

If the drug use and the occurrence of polypharmacy continue to increase, a future challenge for health care will be to treat the resulting side effects. Therefore, the evaluation and reconsideration of the drug therapy, especially for patients who receive drugs via several different doctors, should be a standard procedure before prescribing a new drug. On an individual level, a prescriber seems to need an overview of all the patients medications, including other prescribers and OTC drugs and also earlier dispensed "if needed drugs", to be able to optimize the patient's treatment. Observed multiple medications for an individual should serve as a warning signal, reminding the prescriber that the number of dispensed drugs, together with an uncertain number of drugs from additional sources, may be a risk factor for the patient's health.

### Cost effects

The costs associated with drug-related problems have been estimated to have more than doubled between 1995 and 2000 [36]. Moreover, the risk for drug-drug interactions and adverse drug reactions are expected to increase exponentially with the number of drugs consumed [9]. If the current increase in the total drug consumption and multiple medications will continue, there will be a considerable risk of increased primary as well as secondary costs for drug-related problems in the society. The relationship between the prevalence of multiple medications and the cost for drug-related problems remains to be studied.

## Conclusion

Our study of an entire national population demonstrated that the prevalence of dispensed drugs and multiple medications were extensive in all age groups and were higher for females than for males. Consequently, rational and irrational multiple medications should be regarded as a risk factor in terms of potential drug-drug interactions and adverse drug reactions in all age groups. All sources of medications and generic duplications taken into account, the prevalence of multiple medications would be even greater.

## Competing interests

The authors declare that they have no competing interests.

## Authors' contributions

All authors participated in the design of the study and the discussion of the findings. BH carried out data management and drafted the manuscript. BÅ and GP revised the manuscript. All authors read and approved the final manuscript.

## Pre-publication history

The pre-publication history for this paper can be accessed here:


